# Integrated Microfluidic-Based Platforms for On-Site Detection and Quantification of Infectious Pathogens: Towards On-Site Medical Translation of SARS-CoV-2 Diagnostic Platforms

**DOI:** 10.3390/mi12091079

**Published:** 2021-09-07

**Authors:** Andres Escobar, Phyllis Chiu, Jianxi Qu, Yushan Zhang, Chang-qing Xu

**Affiliations:** 1 School of Biomedical Engineering, McMaster University, 1280 Main Street West, Hamilton, ON L8S 4L8, Canada; escoba3@mcmaster.ca (A.E.); quj@mcmaster.ca (J.Q.); zhang749@mcmaster.ca (Y.Z.); 2 Department of Engineering Physics, McMaster University, 1280 Main Street West, Hamilton, ON L8S 4L8, Canada; chiuc23@mcmaster.ca

**Keywords:** microfluidics, diagnostics, infectious pathogens, SARS-CoV-2, COVID-19, on-site, medical diagnosis

## Abstract

The rapid detection and quantification of infectious pathogens is an essential component to the control of potentially lethal outbreaks among human populations worldwide. Several of these highly infectious pathogens, such as Middle East respiratory syndrome (MERS) and severe acute respiratory syndrome coronavirus 2 (SARS-CoV-2), have been cemented in human history as causing epidemics or pandemics due to their lethality and contagiousness. SARS-CoV-2 is an example of these highly infectious pathogens that have recently become one of the leading causes of globally reported deaths, creating one of the worst economic downturns and health crises in the last century. As a result, the necessity for highly accurate and increasingly rapid on-site diagnostic platforms for highly infectious pathogens, such as SARS-CoV-2, has grown dramatically over the last two years. Current conventional non-microfluidic diagnostic techniques have limitations in their effectiveness as on-site devices due to their large turnaround times, operational costs and the need for laboratory equipment. In this review, we first present criteria, both novel and previously determined, as a foundation for the development of effective and viable on-site microfluidic diagnostic platforms for several notable pathogens, including SARS-CoV-2. This list of criteria includes standards that were set out by the WHO, as well as our own “seven pillars” for effective microfluidic integration. We then evaluate the use of microfluidic integration to improve upon currently, and previously, existing platforms for the detection of infectious pathogens. Finally, we discuss a stage-wise means to translate our findings into a fundamental framework towards the development of more effective on-site SARS-CoV-2 microfluidic-integrated platforms that may facilitate future pandemic diagnostic and research endeavors. Through microfluidic integration, many limitations in currently existing infectious pathogen diagnostic platforms can be eliminated or improved upon.

## 1. Introduction

The rapid detection and quantification of highly infectious pathogens have historically been difficult to properly achieve and manage outbreaks for. The coronavirus disease 2019 (COVID-19), caused by SARS-CoV-2, is one of these highly infectious pathogens, whose outbreaks led to SARS-CoV-2 becoming the third-leading cause of global deaths in children and adults since January 2020 [1]. The World Health Organization (WHO) had officially reported over 4,400,000 deaths caused by SARS-CoV-2 worldwide by August 2021 [2,3]. The infectivity, mortality and often asymptomatic nature of SARS-CoV-2 make this particular infectious pathogen intrinsically difficult to test. Therefore, devices that are capable of fast detection and quantification at a low cost are critically important to patient treatment and outbreak control. Recently, significant effort was made around the world to address this issue through microfluidic integration. As a result, diagnostic testing has advanced rapidly across several fields, leading to the development of novel diagnostic tools for accurately and quickly identifying patients infected with SARS-CoV-2 [4,5].

Polymerase chain reaction (PCR) is a well-established technique that is widely used in medical, molecular biology and synthetic biology settings for amplifying DNA segments. Most recently, reverse transcriptase PCR (RT-PCR) has seen some increased popularity due to its use as a diagnostic mechanism in the detection of SARS-CoV-2 [6]. PCR is a technique that can be used to amplify the desired segment of DNA or RNA, such as SARS-CoV-2 viral RNA [7]. PCR occurs in three major steps: denaturation, annealing and elongation. At denaturation, the original DNA double strand is heated until it splits into two complementary strands at roughly 95 °C before being mixed with DNA primers. Each primer is designed to have the homology of roughly 20 base pairs to one of the two individual DNA single strands (template strands) to ensure specificity [8]. These primers are designed to immediately flank the targeted sequence from both ends to ensure complete replication of the desired region. To ensure proper annealing, the temperature is lowered to about 65–68 °C, whereby the process of elongation is then initiated [8]. DNA polymerase then begins to attach complementary nucleotides (bases) to the bases in each respective template strand in the 5′ to 3′ direction [8]. The use of PCR can facilitate the diagnosis of infectious diseases in a reasonably short amount of time with sufficient accuracy and sensitivity [9].

On the other hand, immunohistochemistry assays take advantage of the specific binding properties of antibodies and aptamers for the detection of biomarkers. Since one antibody or aptamer only binds to one antigen, false positives rarely occur [10]. An antibody or aptamer must be thoroughly tested to maximize efficient binding with the target antigen, often with aptamers demonstrating greater binding affinities [11]. Enzyme-linked immunosorbent assay (ELISA) is a well-known technique in molecular biology, where antigens are bound to the surface and antibodies are applied afterward [12,13]. It is worth noting that in microfluidic immunoassay devices, the antibodies are attached to magnetic beads, which is the opposite of ELISA. However, they work under the same mechanism, where the antibody–antigen binding causes a color change to occur, allowing for the quantification of biomarkers [13,14]. Typically, these immunohistochemistry assays take longer than 24 h to be processed in a laboratory setting and were shown to be quite reliable tools for diagnosing SARS-CoV-2 in patients [15].

Despite the advantages and reliability of the commonly used diagnostic tools, such as PCR and immunohistochemistry assays, there are several shortfalls when it comes to rapid and cost-effective medical diagnosis in the field [16,17,18]. These shortfalls become more pronounced and significant when a high demand for accurate and sensitive rapid testing is not being met due to the high cost and infrastructure barriers. Moreover, to prevent and control the spread of a highly infectious virus, such as SARS-CoV-2, the demand for tests that are quick, accurate and can be widely distributed to communities becomes enormous. Commonly used SARS-CoV-2 diagnostic methods, such as RT-PCR and immunoassays, lack several features that would make them a widely accessible tool that is capable of meeting the pandemic-fueled demand for reliable on-site medical diagnostic tests [10,12,19]. The most important features of these on-site medical diagnostic tests include fast detection, quantitative detection, high sensitivity and low cost. Currently, commonly used tests are only capable of reliably diagnosing patients as early as two weeks after the initial infection with an accuracy between 70–90%, using detection limits that can often be quite low (1 DNA/RNA copy per milliliter of transport volume) and can vary significantly in their sensitivity [20,21]. PCR tests were recently showing some promise in terms of faster turnaround times and reduced costs. However, PCR tests produced at a large scale are still often quite expensive, as they usually require lab-scale equipment to process the tests and accurately analyze the results [18,22]. This leaves large gaps in testing requirements, as SARS-CoV-2 requires accurate, accessible and reliable diagnoses within the first 10 days of infection to prevent massive outbreaks [23]. Similar to RT-PCR tests, antibody assays that are used to diagnose SARS-CoV-2 lack the modularity that is required to accurately detect and diagnose the rapidly emerging variants of SARS-CoV-2. There are several other reported methods for medical diagnoses, such as nanoparticle-based detection and cell-culturing techniques, but these techniques do not meet the testing properties required to meet market demand for rapid, accurate and cost-effective tests for medical diagnostic tools [24].

In this review, we focus on the classification of microfluidic devices, the necessary properties of a microfluidic device that define an integrated microfluidic device, examples of partial and fully integrated microfluidic devices for the detection of infectious pathogens and examples of SARS-CoV-2 diagnostic technology with the potential for clinical on-site translation. The goal of this review was to provide a comprehensive understanding of the currently available diagnostic tools and how microfluidic integration can facilitate the clinical translation of lab-scale research toward on-site diagnostics of SARS-CoV-2. As our review focused on infectious pathogens, the mechanisms of detection that are discussed are mainly nucleic acid amplification tests (NAAT) and immunoassays, as they are in closest relation to bacterial and viral pathogens. Other mechanisms of detection for biomedical applications, such as cell sorting, solid-phase extraction (SPE) and nanoparticles, are not discussed in detail in this review but may be more appropriate depending on the intended detection target [25,26,27]. This review presents a foundational framework by which current and future research for the detection and quantification of infectious pathogens, including SARS-CoV-2, can increase the viability and effectiveness of their diagnostic platforms for on-site use through microfluidic integration.

## 2. Integrated Microfluidic-Based Platforms (IMPs)

### 2.1. Classification of IMPs

Integrated microfluidic platforms (IMPs) refer to diagnostic or analytic platforms that utilize, in part, a microfluidic-based system to facilitate the combination of several different steps of medical diagnoses toward full or partial automation [28,29,30]. The integration of microfluidics in novel diagnostic technologies takes advantage of micrometric-scale fluids behaving very differently than fluids at volumes seen in our everyday lives. The two main types of currently existing microfluidic systems are continuous-flow and droplet-based systems that can both be further classified based on their respective materials and detection methods. Droplet microfluidic systems produce a large quantity of distinct fluid microenvironments that are suspended in a separate immiscible phase, with each acting as independent miniature bioreactors [31]. In droplet microfluidics, individual droplet microenvironments of discrete volumes are formed through the controlled, segmented flow of various reagent-containing fluids and encapsulating them in an immiscible medium to form the individual droplets [31]. This contrasts with continuous-flow microfluidic systems, which utilize steady-state liquid flow through narrow channels or porous media to introduce necessary samples to reagents and form one homogenous mixture. Through microfluidic integration, either of these main types of microfluidic systems can offer their own respective advantages and disadvantages to a previously non-integrated diagnostic method.

Both types of microfluidic systems can be leveraged as a complementary component to existing diagnostic tools to attain complete or partial automation, multiplexing and high throughput [32,33]. These medical diagnosis steps generally include sample preparation, sample collection and labeling, signal reading and data analysis. 

**Figure 1:**
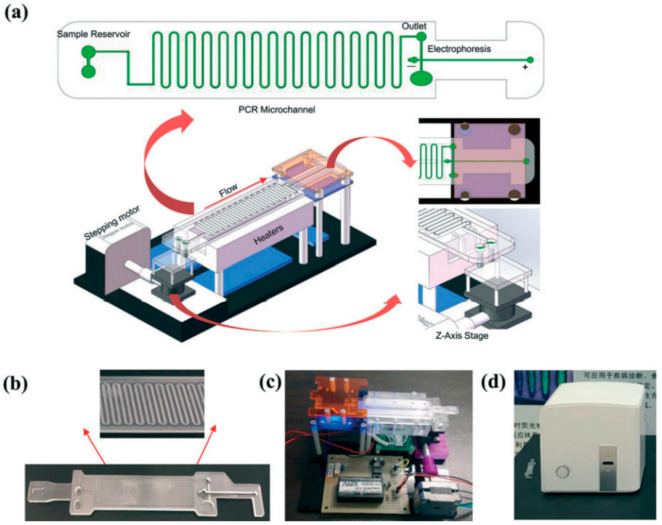
A schematic workup of an integrated microfluidic device that is used for pathogen diagnosis using a PCR-based technique. (**a**) A microfluidic device that will be incorporated with other electronic components to automate the diagnostic process. (**b**) The physical microfluidic chip is built based on the initial design. (**c**) The prototype of the integrated microfluidic device. (**d**) The final “all-in-one” version of the integrated microfluidic device. Reprinted with permission from ref. [34]. Copyright 2019 Royal Society of Chemistry.

Figure 1 shows a schematic workup of a continuous-flow integrated microfluidic device used for pathogen diagnosis using a PCR-based technique [34]. Continuous-flow microfluidic devices can generally be further classified through the shape of their microchannels. The four main sub-types of continuous-flow microfluidic devices include serpentine, spiral, oscillating-flow and straight microchannels [9]. As shown in Figure 1, the need for multiple trained operators, external reagent preparation and laboratory-scale equipment was removed in this serpentine continuous-flow microfluidic device [34]. In addition, this integrated continuous-flow microfluidic device is fully automated and can process the data collected from the sample in real time. The network of microchannels can be designed and tailored to fit the needs of the desired analysis and can offer several different advantages compared to conventionally sized systems. Droplet microfluidics, the second main type of microfluidic system, can offer similar improvements to modularity and automation to a continuous-flow system compared to non-integrated microfluidic systems.

**Figure 2:**
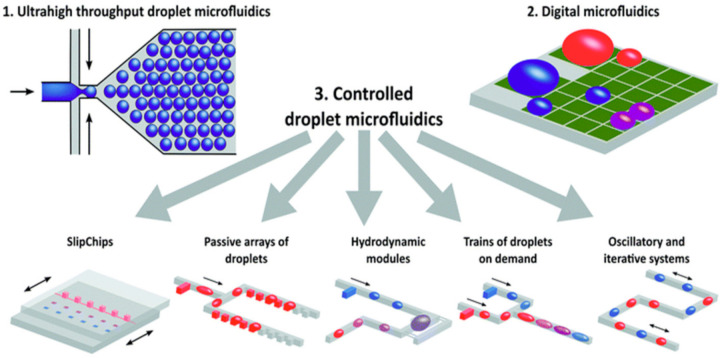
A visual summary of several key forms of droplet microfluidics. Reprinted with permission from ref. [31]. Copyright 2017 Royal Society of Chemistry.

As shown in Figure 2, droplet microfluidics can be applied to a variety of different operational mechanisms; thus, they offer their own type of subclassifications [31]. In the case of droplet microfluidics, as shown in Figure 2, the three main sub-types of droplet microfluidic devices are ultrahigh throughput, digital and controlled droplet microfluidics [31]. Figure 2 offers a useful visual representation of the versatility that is achieved with a droplet microfluidic platform and effectively demonstrates the mechanistic goals of each sub-type [31]. However, droplet microfluidics is often tailored toward extremely high throughput and single-cell tasks, which cannot be readily achieved through their continuous-flow counterpart. Moreover, droplet microfluidic platforms are more capable of high-throughput assays through the production of a high number of contained microenvironments, which is a feat that is extremely difficult to achieve through continuous-phase microfluidic systems. These differences are further explored in this review.

Both continuous-flow and droplet microfluidics can be further categorized and classified by either the type of material used to build the device or by the mechanism of detection [35]. Based on the type of material used, the classification of microfluidic devices typically falls under one of four kinds: glass, silicon, polymer and paper [36]. Each of these types of microfluidic devices can be fabricated in different ways, at different costs and each presents its own unique combination of advantages and disadvantages [35]. Depending on the goal of the microfluidic device being integrated into a diagnostic device, one can select the material of microfluidic device that best suits their needs. In addition to the classification by material, microfluidic devices can be classified by the detection mechanism, such as PCR, nanoparticles, antibodies, aptamers, molecular diagnostics and smartphone-based diagnostics. This review mostly focuses on the assessment of PCR-based and immunoassay-based diagnostic techniques that demonstrate potential for the detection and quantification of infectious pathogens through the integration of microfluidics.

### 2.2. Current Research Trends Guiding the Development of IMPs

The integration of microfluidics in different forms of analysis can reduce global operational costs of certain processes in both laboratory and in-field environments. Several forms of analysis that are facilitated by the integration of microfluidics include, but are not limited to: DNA amplification, single-cell analysis and the detection of targeted genetic material. Due to the modularity and microscale of microfluidic technology, several of these processes can often be run in parallel, minimize the sample and reagent volumes required to operate these processes and greatly reduce turnaround times through process automation. Process automation can, in turn, reduce the need for multiple trained operators to safely handle, transport and analyze samples used in the integrated microfluidic systems. Moreover, through microfluidic integration, the ability to mass-produce these analytical systems at a consistent level of quality will afford researchers increased testing capabilities with minimal expense to the quality and accuracy of the processes. These platforms can become an asset in almost any type of research that involves fluid control; however, there are several potential microfluidic applications of note, such as point-of-care (POC) and on-site (OS) diagnostics. The reduced global cost and increased number of features offered by microfluidic integration made microfluidic platforms an increasingly popular medium for the detection of infectious pathogens in human samples, such as SARS-CoV-2.

The advent of the COVID-19 pandemic has, in part, driven the increased demand for improved on-site microfluidic diagnostic systems as a means to better identify, monitor and control infected patients in the hopes of preventing mass outbreaks. On-site diagnostic tests refer to biological analyses and tests that can be carried out and processed in resource-limited environments or environments with no access to medical laboratories [34]. On-site integrated microfluidics-based platforms currently hold the most relevance and importance as SARS-CoV-2, and its more infectious variants, continue to spread and be one of the most prevalent causes of deaths around the world. Due to their modularity, reproducibility and increased sensitivity, IMPs are capable of better facilitating the diagnosis of patients with infectious pathogens, such as SARS-CoV-2, more reliably and earlier compared to commonly used diagnostic methods lacking microfluidic integration [37]. In addition, microfluidic platforms can act as closed-reaction platforms, which increases the accessibility of these diagnostic tests by reducing the risk of contamination to handlers and facilitating transport. Therefore, integrated microfluidic-based platforms, as an essential component of infectious pathogen diagnostic testing, holds great potential for the advancement of medical diagnostics and research in both clinical and on-site practices.

### 2.3. Criteria for Assessing IMPs

#### 2.3.1. WHO On-Site Diagnostic Device Standards

The World Health Organization (WHO) describes the criteria necessary for determining whether a microfluidic-based diagnostic device qualifies for on-site (OS) or point-of-care (POC) use [5,38,39]. The diagnostic devices must be “affordable, sensitive, user-friendly, rapid and robust, equipment-free and deliverable to end users”, otherwise known as ASSURED. The ASSURED criteria can, therefore, be used to assess the efficacy and applicability of integrated microfluidic-based currently existing or future diagnostic devices prior to mass production and mass distribution.

Many well-established conventional non-microfluidic integrated diagnostic techniques can struggle to address the first ASSURED criteria of affordability due to their diagnostic techniques often requiring costly reagents, fabrication material, knowledgeable operators and laboratory-grade equipment [40,41]. Microfluidic integration could greatly improve affordability by reducing global costs related to reagent volumes and fabrication materials through the size reduction of diagnostic systems from the lab scale to the microscale. Smaller reagent volumes per test lead to a decreased cost per test and can increase the number of tests possible per unit volume of reagent. The size reduction to the microscale would also greatly reduce the amount of material required to fabricate each test and increase its affordability.

Continuous-flow microfluidics and droplet microfluidics can both be integrated to achieve increased sensitivity through system design features that would facilitate improved sample–reagent interactions [9]. For example, non-microfluidic diagnostic platforms might stray away from certain reagents with greater binding affinity to target molecules due to the large reagent volumes that would require greater costs. However, in microfluidic integrated systems, reducing the reagent volumes that are required to operate a test may offer more effective diagnostic marker alternatives compared to their non-integrated counterparts. Moreover, the technology available for analyzing patient samples at the microscale is greater than those available for analysis at the lab scale. Therefore, the modularity offered by microfluidic integration can help to address the sensitivity criteria set out by the WHO.

Microfluidic systems can, and are often, designed to serve as self-contained bioreactors in order to precisely analyze patient samples and reduce the potential influence on results from external factors. This design principle can be applied to both continuous-flow and droplet microfluidics in different scales [37]. Droplet microfluidics can produce thousands of discrete microvolume bioreactions that are surrounded by an immiscible phase, whereas continuous-flow microfluidics is often used to process patient samples in a single bioreaction contained by the physical device [31]. By being self-contained, both the patient being tested and the operator processing the sample are exposed to fewer safety risks and can reduce the number of unnecessary complications in translating the results to researchers and patients alike. In addition, many of the currently available POC diagnostic tools utilize methods such as PCR, ELISA and other antibody immunoassays that involve a great deal of sample preparation steps and are time consuming. Microfluidic integration aims to combine and automate several of these essential sample-processing steps in order to minimize the turnaround time and increase the robustness of each test. Therefore, microfluidic integration could help to overcome the limitations of sensitivity, user-friendliness, rapid-testing and device robustness, in line with the ASSURED criteria, and drive forward the development of commercially available and cost-effective diagnostic devices.

Device cost and performance standards are not the only necessary criteria by which on-site diagnostic devices need to be assessed. On-site devices for medical diagnosis require the least amount of equipment to operate and require effective delivery methods to end users. Through microfluidic integration, self-contained and automated diagnostic tools would help to minimize the amount of equipment needed to operate the tests while also facilitating the transportation, storage and operational safety of the tests to end users. Due to the high demand for safe, readily available and cost-effective diagnostic tests for a highly infectious and potentially lethal virus, the need for on-site integrated microfluidic-based diagnostic tools has never been greater.

The ASSURED criteria serve as an effective list of fundamental criteria to assess the viability of current and future diagnostic tests and can be addressed through microfluidic integration of either continuous-flow or droplet microfluidic systems. However, these criteria should not be the only means by which researchers should critique and evaluate the effectiveness and viability of diagnostic tests. Therefore, through this review, we present additional criteria for the validation of current and future on-site diagnostic tests.

#### 2.3.2. Pillars for Assessing Effective On-Site Diagnostic Tools

The WHO developed the ASSURED criteria to evaluate the validity of on-site and point-of-care diagnostic tests; however, these criteria alone do not evaluate the effectiveness and quality of those tests as strongly as they should. In response, we have identified seven pillars that address the most essential issues pertaining to diagnostic tools, which together have formed novel standards for the current and future research and development of integrated microfluidic devices to adhere to. 

**Figure 3:**
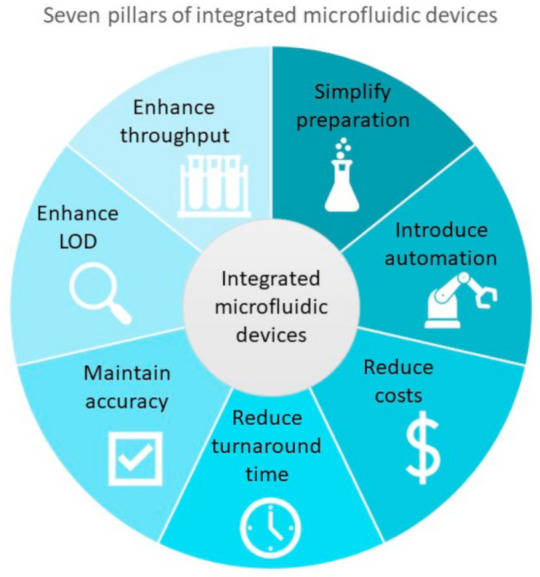
Seven identified pillars of integrated microfluidic devices including: simplified preparation, automation, cost reduction, turnaround time reduction, maintained accuracy from conventional counterparts, enhanced limit of detection and enhanced throughput.

The pillars shown in Figure 3 include: simplify preparation, introduce automation, reduce costs, reduce turnaround times, maintain accuracy, enhance the limit of detection (LOD) and enhance the throughput. Although we have separated the criteria into seven distinct pillars, they are in fact intertwined and often influence one another. Achieving at least one of the seven pillars will undoubtedly improve diagnostic processes and enable the transition from centralized testing to POC and OS detection.

As previously mentioned, the main goal of an integrated microfluidic device is to facilitate the combination and automation of several different steps in the process of diagnosing patients. However, there are more factors involved in designing and producing an effective integrated microfluidic device. Therefore, these seven pillars will serve as enhanced criteria for developing effective microfluidic diagnostic devices for infectious diseases and should be closely followed.

These enhanced criteria can often prove difficult to address in conventional non-microfluidic devices; however, the advantages offered by microfluidic integration will undoubtedly facilitate the achievements of many of these pillars for effective diagnostic tests of infectious diseases.

### 2.4. Advantages to Microfluidic Integration

Microfluidic integration can offer a variety of advantages to diagnostic testing platforms that simply cannot be mimicked in conventional diagnostic tests. Therefore, technological advancements toward the identification and quantification of infectious pathogens, such as SARS-CoV-2, must consider microfluidic integration.

#### 2.4.1. On-Device Sample Preparation

Diagnostic tools often require some sort of sample preparation to remove undesired material that may interfere with the accuracy and sensitivity of test results. By considering the seven pillars of integrated microfluidic devices in the design process, as shown in Figure 3, and integrating microfluidics, one could simplify the required sample preparation steps and create a continuous reaction that is performed and contained within the device.

For example, nucleic acid (NA) extraction is required for most amplification methods, such as PCR. In the detection of pathogens, thermal lysis is usually employed so that bacteria and viruses release their genetic material, enabling the amplification of such genetic sequences. The reported temperature for bacterial cell lysis is between 95 and 103 °C and 95 °C for viral lysis and can be easily achieved through the integration of microfluidics [42,43,44]. By integrating a digitally controlled heating unit or by heating the microfluidic chip itself, the thermal cycling processes required for PCR can be tracked and externally controlled. Alternatively, using a lysis solution (2% *v/v* xylene, 7.9% *v/v* acetone, and 0.1% *v/v* toluene) can facilitate the isolation of genetic material by breaking down cellular components, such as the cell walls, to release the contents of each cell [45]. In fact, chemical lysis may be preferred for isothermal amplification methods, such as LAMP, due to its sensitivity to heat, thereby eliminating the need for a thermal cycler for the lysis step.

Preconcentration is another step that is related to the sample preparation that is used to facilitate sufficient target–reagent interactions necessary for effective diagnostic analysis. This preconcentration step is often used in the treatment of protein biomarkers, as they cannot be amplified, unlike nucleic acids. One way of achieving preconcentration is through protein adsorption with a porous membrane. Mohamadi et al. presented a microfluidic device that uses a polyethylene glycol diacrylate (PEG-DA) membrane with nanometer-sized pores in the preconcentration of amyloid-β peptides in cerebral spinal fluid for the diagnosis of Alzheimer’s disease [46]. This on-chip integration of the preconcentration step is faster and simpler than its off-chip counterpart, as additional equipment is not required for the adsorption process and the trapped peptides can be used in immunocapture immediately after this step [46]. Not only does the implementation of suitable on-device sample preparation steps save time and resources but it can also enhance the performance of the assay, such as improving the LOD of the assay.

#### 2.4.2. Increased Automation

Automation can be implemented with microfluidic integration and facilitate the diagnostic procedure by reducing the turnaround time and improving the replicability of each trial and assay [42,47]. Through automation, the need for manual operation, technically trained operators, constant reagent supply and expensive equipment is heavily mitigated. Ruan et al. demonstrated the effectiveness of automation through a fully automated electrode-controlled procedure to dispense uniform phosphate-buffered saline droplets for the detection of DNA methylation [48]. Thus, the variance that might have resulted from human error was effectively removed and the reliability and reproducibility of this assay were increased. Automation can significantly reduce the number of operators and the need for expensive laboratory equipment; however, it may not be the only type of cost that needs to be considered.

Blood plasma is a commonly used sample medium in diagnostic tests, as it contains various biomolecules as targets for detection. To obtain blood plasma, whole blood is centrifuged to remove the formed elements in the blood. Due to the nature of centrifugation, such as moving parts in equipment and the requirement to achieve high rotational speeds, it is difficult to integrate this step into microfluidic devices. Hence, the focus has shifted to magnetic separation, also known as magnetophoresis, where cells are driven through microfluidic devices using magnetic forces [49]. This passive technique is used primarily by cell-sorting microfluidic devices that detect circulating tumor cells (CTCs) in whole blood, which are indicators of possible cancer metastasis but can also be applied to sample preparation steps [50,51,52,53,54]. As CTCs occur at a very low abundance, about one to ten per billion peripheral blood cells, it is imperative that there is a way to isolate CTCs from the extremely large pool of cells. Lee et al. used magnetized nanoparticles to bind with white blood cells, then used a magnetic cell sorter to remove the interfering white blood cells from the sample, subsequently allowing them to detect one to three CTCs in adjuvant breast cancer patients [51]. Magnetophoresis is an automated process that can be applied as a potential sample preparation step to filter out the interfering cells or large structures present in samples in an IMP.

#### 2.4.3. Cost Reduction

Commonly used diagnostic tests require expensive and complicated laboratory equipment to reliably diagnose patients but this has erected a financial barrier between low resource communities and their access to these tests. In the wake of the COVID-19 pandemic, this financial limitation to low-resource communities, where the demand for tests has driven up the price per test and has left low-income individuals vulnerable, is quite evident. It was found that a COVID-19 test can cost anywhere from 20 to 1419 USD per test in the United States, not including additional fees that may arise from provider visits, facilities fees or collection fees [55]. While these prices alone are not indicative of the cost of a SARS-CoV-2 PCR test itself, the unmet need to reduce the operating costs and the cost of raw materials is still very high. With integrated microfluidic devices, there are several different areas where better design and ingenuity can lead to an even greater reduction in costs.

One area where costs can be reduced is in the choice of materials that are used in the fabrication process of microfluidic devices. Silicon and glass were among the first types of material used to produce microfluidic chips but were eventually determined to be less cost-effective than other alternatives when being mass-produced, due to their initial high fabrication costs [56]. Consequently, paper and polymer-based microfluidic devices have become widely popular due to their low raw costs and simple fabrication techniques, as shown in Table 1, but can often be paired with other materials as needed.

Paper-based microfluidic devices, also known as μPADs, were first developed by Whitesides et al. to push the potential of low-cost microfluidic devices [56]. They used chromatography paper and patterned it with SU-8 photoresist, forming areas of hydrophilic paper that were separated by hydrophobic lines [56]. Liquid is drawn into the microchannels via capillary action, while solid contaminants, such as dirt and pollen, are not drawn into the assay chambers and therefore cannot interfere with the assays [56]. Since then, μPADs have been extensively researched and their performance, as well as their complexity, has improved. Wax printing is now a commonly used fabrication technique in μPADs, where wax is deposited onto both sides of the paper then quickly melted to form hydrophobic channels [58]. Carrilho et al. reported that 100–200 μPADs can be fabricated on an 8.5 in. × 11.5 in. piece of paper within five minutes, with each μPAD costing as low as 0.001 USD if inexpensive paper is used, demonstrating the scalability of μPADs and their propensity to be used in POC and OS detection [59].

Another material that uses non-photolithographic fabrication methods is polystyrene sheets, known to many as “Shrinky-dink” sheets [60,61]. Khine et al. first took leverage of the shrinking properties of commercially available “Shrinky-dink” sheets that were marketed as a children’s toy [60]. Heating the prestressed sheets at 160 °C for three to five minutes results in isometrical in-plane shrinkage by 50% and a 700% increase in the height of the sheet [61]. Although features could be manually scribed onto the polystyrene sheets using tools, such as syringe needles and razor blades, other automated methods, such as a CO_2_ laser cutter, can be used [60,61]. One important advantage of using polystyrene sheets for microfluidic devices is that 3D microfluidic devices can be easily assembled through irreversible thermal bonding of unshrunk sheets, then the stack is shrunk together so that the etching on each layer come together to form 3D features [60]. The shrinking process only requires heat, and a temperature of 160 °C can be easily achieved by a common electrical appliance, such as a toaster oven.

The term “microfluidic” already implies the manipulation of reagents of small volumes, with many agreeing that this ranges from picoliter to microliter levels. By comparison, a standard PCR protocol handles reagents at the microliter and milliliter levels, coinciding with the upper limit of the volume allowed by microfluidics [62]. In general, it can be assumed that all microfluidic devices would reduce the reagent volume. Smaller volumes of reagents per test reduce the cost per test and allow for the redistribution of resources so that more tests can be performed since widespread availability is integral in diagnostic detection.

#### 2.4.4. Shortened Turnaround Time

Time is of the essence in medical diagnostics and in the case of acute diseases and infectious pathogens, the rapid confirmation of a disease allows healthcare providers to treat patients accordingly and help to stop the spread of infectious pathogens [63]. In chronic illnesses, the monitoring of certain biomarkers over time can be reflective of the patient’s recovery or treatment. For many integrated microfluidic devices, tests have been reported to be complete within one hour, with some devices completing the test in only 10 min [63,64,65,66]. The shorter turnaround time can be combined with shorter detection times to maximize the speed at which results can be obtained from these tests. The use of preconcentration steps and microfluidic droplets increases the concentration within the reaction unit, and thus increases the rate at which the reaction occurs. The improvement in assay time when using microfluidic devices is evident when compared to their conventional counterparts. Ye et al. developed an RT-PCR microfluidic device that was used in the detection of perinatal group B streptococcus in pregnant women and reported an assay time of 45 min, which is less than half the 2–3 h that is needed in a conventional RT-PCR assay [44].

Detection time can also be truncated through simple assay results. This is typically achieved by chemiluminescence and colorimetric assays, where results may be binary in nature and obvious enough that they can be observed with the naked eye, eliminating the need for processing and analyzing software and extensively trained personnel to interpret the results [67]. In a review on μPADs by Tian et al., they found that the vast majority of μPADs, which are usually employed in POC and OS detection, use chemiluminescence and colorimetric assays that are easily interpreted [68]. Maximizing the number of controllable variables, through improved detection time, will effectively minimize the interference of these variables with the results, and therefore, increase the reliability and accuracy of the test results.

#### 2.4.5. Maintaining the Level of Accuracy Seen in Conventional Counterparts

Many microfluidic devices scale down well-established assay methods instead of implementing new techniques. Due to this feature, it is easier to apply current methods onto microfluidic platforms and it provides a benchmark for the accuracy levels that the platform should attain. This ensures that the reliability of conventional tests is represented in their microfluidic adaptations and can be used as trusted alternatives. A microfluidic platform developed by Zhu et al. was used for detecting five pathogenic genotypes of the human papillomavirus (HPV) and the results from 20 different samples were compared to a standard qPCR assay [69]. Admittedly, this is not a direct comparison as the device reports qualitative results while the qPCR assay reports quantitative results, but the device is still able to accurately detect the presence of HPV in the samples [69]. Another platform designed by Fu et al. is used to perform immunoassays on three highly contagious swine pathogens [70]. The reported accuracy rates range from 93.8 to 96.8% [70]. This falls short of the specificity of 100% reported for conventional ELISA performed on swine viruses [71]. However, it should be noted that sensitivity decreases with lower viral loads, with an average of 57% across different viral loads [71]. Leveraging microfluidic platforms to improve the LOD can mitigate this problem at lower viral loads, as Fu et al. reported a sensitivity of at least 88% for their device [70].

#### 2.4.6. Improving the Limit of Detection (LOD) of Assays

Biomarkers that do not exist in sufficiently large quantities in bodily fluid samples, such as those for cancers and certain neurodegenerative diseases, have the potential to evade detection in conventional diagnostic tests. Due to the low sensitivity of these conventional diagnostic tests, detection issues that are related to low biomarker abundance remain a problem that can be readily observed in protein biomarkers, as they cannot be duplicated by conventional amplification methods. As a result, microfluidic-based solutions, such as automated droplet production, can assist in the detection process. The small volume of microfluidic droplets, usually ranging from femtoliters to nanoliters, can be leveraged in the detection of low abundance biomarkers, where essentially one reaction molecule occupies one droplet. This increase in the relative concentration of the target molecule within the droplet will allow for the detection test to zero in on the target, making it easier to detect this molecule, which may otherwise be overlooked in a standard assay. Shim et al. presented a microfluidic device that used bead-based ELISA in the detection of prostate-specific antigen (PSA) for prostate cancer. They were able to detect PSA in concentrations as low as 46 fM, and conclusively detected PSA at a level that would not have been detected in standard ELISA [66]. Droplets containing a copy of the target genetic material will have the target genetic material replicated, tagged by a fluorophore, identified by fluorescence imaging and labeled as a positive droplet. Conversely, negative droplets do not initiate any replication reaction, as the target genetic material is not present. This simplified and self-contained droplet reaction analysis allows for the quantification of targeted DNA present [72]. By integrating droplet-focused microfluidics and RT-qPCR, these techniques can be used to detect low levels of expressed genes in a manner that would reduce global costs of operation and require smaller reagent volumes. As a proof of concept, Hajji et al. demonstrated the use of integrated microfluidics and droplet RT-qPCR to detect the overexpression of the HER2 gene in breast cancer patients, where the quantification of low-abundance RNA is necessary to determine whether overexpression has occurred [73].

#### 2.4.7. Integrating High-Throughput Assays

The miniaturized equipment used in devices increases the portability of the entire apparatus, allowing for the transition from a traditional laboratory setting into an independent operating unit, which can be used as handheld devices in low-resource settings. An added benefit of miniaturization is that the microfluidic chips can be made so that they perform high-throughput assays. This is achieved through densely packing well chambers or flow strips so that tests can be run in parallel [74]. Many integrated microfluidic devices have testing units that can be easily replicated to fulfill parallel testing on the same device [69]. Parallel testing in conjunction with shortened turnarounds times will undoubtedly boost the testing efficiency to unprecedented levels and set new benchmarks for diagnostic tests.

Microfluidic integration has been demonstrated to show improvement in the throughput of several different types of assays. High-throughput droplet microfluidic assays appear to show the most promise towards infectious pathogen diagnostics [75]. Through the formation of thousands of controlled discrete microvolume bioreactor environments, techniques such as antibody screening were exponentially improved through microfluidic integration.

## 3. Using IMPs in the On-Site Detection of Infectious Pathogens

The unmet need for diagnostic tools to detect respiratory viral infections, namely, SARS-CoV-2, is not the only diagnostic effort that can benefit from microfluidic integration. Reliable diagnostic tools that are aimed at readily detecting and quantifying other non-respiratory viral infections in patients of low-resource areas are required to effectively track and manage the possible spread of the disease.

### 3.1. Partially Integrated IMPs in the On-Site Detection of Infectious Pathogens

The Zika virus (ZIKV) is one example of a pathogen that can be transmitted by infected mosquitos and bodily fluid exchange that can cause symptoms in humans, such as fever and headaches, as well as microcephaly in newborn babies [76]. It was demonstrated how a nucleic acid amplification test (NAAT) was able to be performed with a partially integrated microfluidic device, developed by Kaarj et al., with a shorter turnaround time than conventional non-microfluidic-based tests [77].

Reverse transcriptase isothermal lamp mediated amplification (RT-LAMP) is an alternative amplification technique to PCR that can be used to similarly amplify target genetic material, up to 10^−9^ copies per hour, performed under isothermal conditions [78]. The complementary properties of the primers and DNA sequences form self-hybridizing loops in the amplification products, which have annealing sites to initiate the next round of amplification [78]. Due to the simplicity of its operation, LAMP is commonly chosen for μPADs, as it does not require thermal cycling and is more appropriate for POC and OS diagnostic detection [79].

**Figure 4:**
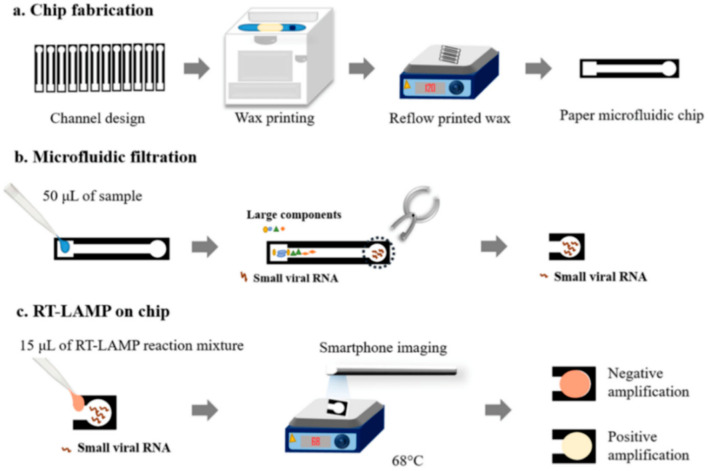
The components to the processes of (**a**) chip fabrication, (**b**) microfluidic filtration and (**c**) RT-LAMP on the chip. In addition, it demonstrates a self-contained integration of a microfluidic platform to facilitate detection via a smartphone. Reprinted with permission from ref. [77]. Copyright 2018 Springer Nature.

The paper microfluidic chips shown in Figure 4 were designed using SolidWorks and were wax printed on cellulose grade 4 paper, which was shown to have a relatively fast turnaround time and heightened amplification compared with conventional non-microfluidic integrated diagnostic tests [77]. The device operates under capillary action, where the samples (deionized water, tap water, human urine and human blood plasma) are deposited into the loading area and drawn into the channel [77]. The paper-based platform also acts as a filter for larger contaminants in the samples as they cannot move through the channel and will not end up in the detection area [77]. Once the analyte is fully contained in the detection area, that detection area will be excised and then added to the RT-LAMP reaction mixture containing primers specific to the NS5 gene in ZIKV to facilitate amplification at 68 °C [77]. The detection area is sandwiched between two pieces of glass to prevent evaporation. The presence of ZIKV is reported through the colorimetric pH indicator phenol red in the reaction mixture, which is measured using raw RGB intensities. As the amplification progresses, the paper changes color from a yellowish-red (red + weak green) to yellow (red + green) [77]. By measuring each of the raw RGB intensities at various times, the progression of the assay can be monitored, and any anomalies can be detected [77]. The analysis of the red-green-blue (RGB) color model intensities can be done with a smartphone camera in ambient lighting, once again, highlighting the importance of partial microfluidic integration in facilitating rapid, reproducible, cost-effective and accurate paper-based diagnostic testing [77].

The detection limit and accessibility of a diagnostic test can also be improved through the partial integration of microfluidics. For example, the limit of detection for this ZIKV-specific device was found to be 1 copy/μL [77]. It should be noted that despite the low limit of detection, lower concentrations (1–10 copies/μL) show an uneven distribution of the yellow coloration and yield greater inaccuracies that are not present in higher concentrations (more than 100 copies/μL) [77]. Interestingly, this device excels in the sample preparation and cost reduction pillars. The ability of the microchannels to filter out unwanted contaminants replaces any need for external machinery to assist in the sample preparation steps [77]. The materials and fabrication method of the μPAD is also cost-effective and can be easily scaled up to be mass-produced [77]. The use of a smartphone camera for colorimetric detection also increases the accessibility of this diagnostic test and allows this assay to be completed in 15 min, making it suitable to be used in rapid testing for ZIKV [77].

While there are no automated steps for this particular type of μPAD, an argument that this is redundant can be made. The manual steps in the assay include excising the detection area, adding the RT-LAMP reaction mixture and placing the paper between two glass slides, all of which are not very technical steps. Therefore, introducing some level of automation into this assay may decrease the portability and ease of use of the device. Despite the device not demonstrating high-throughput detection, the operation is simple enough that many samples and assays can be handled at once.

The ZIKV paper-based microfluidic platform serves as a basis for the microfluidic-based detection of viral pathogens, utilizing aspects such as sample preparation and nucleic acid amplification. ZIKV belongs to the Flaviviridae family and has positive-strand RNA as its genomic material, which can be directly used in RT-LAMP [80]. Similarly, SARS-CoV-2, which belongs to the Betacoronavirus family, has positive-strand RNA as its genomic material, making it a suitable target to be used in RT-LAMP [81]. RT-LAMP is a technique that was already explored for SARS-CoV-2, with Thi et al. reporting a quantitative RT-LAMP colorimetric assay [82]. Similar to the ZIKV device, they used a colorimetric pH dye phenol red as the reporter of the assay, which was already demonstrated to be compatible with the RT-LAMP reaction mixture by Kaarj et al. [77,82]. Therefore, choosing RT-LAMP as the mechanism for SARS-CoV-2 NAAT is a viable option and can be implemented onto an IMP.

### 3.2. Fully Integrated IMPs Developed for the Detection of Infectious Pathogens

Fully integrated microfluidic platforms refer to devices where most, if not all, steps of the diagnostic test are integrated onto one single device and require little to no manual operation. This attribute is especially beneficial in microfluidic devices used in the detection of infectious pathogens, as the simple operation of the device allows it to be widely distributed into communities for testing.

**Figure 5:**
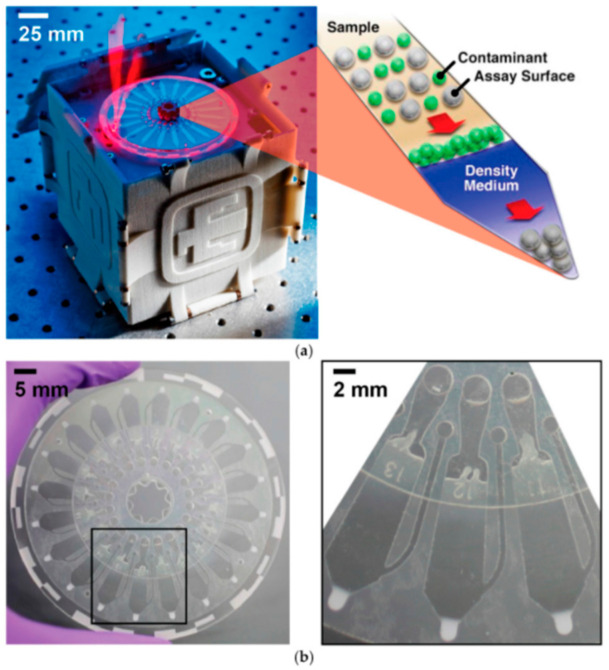
(**a**) Microfluidic platform performing epifluorescence detection (left) and the principle of sedimentation-based immunoassay (right). (**b**) Lab-on-a-disc device with 20 microchannels (left) and the detailed architecture of the microchannels showing the concentrated pellet of beads at the end of the channels (right). Reprinted with permission from ref. [84]. Copyright 2016 MDPI.

With enteric and diarrheal diseases causing 8% of the deaths in children under the age of five, it is imperative that a reliable testing system can be launched to avoid these preventable deaths [83]. Unlike the previous ZIKV device, which detects one viral pathogen, this device developed by Phaneuf et al., shown in Figure 5, is capable of the multiplexed detection of four bacterial pathogens that cause enteric diseases [84].

This lab-on-a-disc device uses a centrifugal sedimentation immunoassay to report the presence and quantity of *E. coli, Listeria, Salmonella* and *Shigella* in stool, urine and blood samples [84]. As shown in Figure 5, the disc is made of two layers of poly(methyl methacrylate) (PMMA), with 20 identical assay channels etched using a CO_2_ laser cutter and subsequently joined with a pressure-sensitive adhesive (PSA) [84]. Samples including whole human blood, human saliva, human urine, mouse serum and mouse feces were incubated with magnetic beads conjugated with prelabeled polyclonal antibodies of the four pathogens, then loaded onto the disc [84]. Using a computer-controlled prototype device, the disc is spun at 8000 RPM and the channels were analyzed through laser-induced fluorescence [84]. Relative fluorescence values were then reported to a connected computer for further analysis. The device could complete this immunoassay within 30 min, with a limit of detection (LOD) ranging from 10 to 1700 cells of bacteria from a 1 μL sample for the four bacterial pathogens [83]. Both the singleplex and multiplex operations of this device showed an improved LOD compared to a standard ELISA protocol [84].

This device meets the sample preparation pillar, only requiring serial dilution of the samples and pipetting into the disc [84]. Compared with other laboratory techniques, serial dilution is a simple operation that untrained personnel can master easily. Moreover, the device is fully automated after the prepared samples are pipetted onto the disc. The architecture of the microchannels was designed to take full advantage of the sedimentation-based immunoassay [84]. This is shown in Figure 5, where the centripetal force of the device forces the formed pellets into the periphery of the disc for data analysis [84]. Together, the minimal preparation and the automation increase the ease of use of the device, allowing it to be widely distributed for use.

The device outcompetes its ELISA counterpart in both the assay time and LOD pillars. The sedimentation immunoassay takes 30 min to complete, which is only one-quarter of the time required for an ELISA [84]. As discussed earlier, the LOD of all four bacterial pathogens is improved significantly across all the tested samples, thereby increasing the sensitivity of the assay [84].

An additional benefit of this device is its flexibility in the sample source. With singleplexed assays performed on all samples and a multiplexed assay performed on stool samples successfully, the unparalleled versatility showed by this device eliminates the limitations of the samples [84]. Therefore, the sample source with the best LOD for a specific bacteria can be chosen to improve the testing efficiency. For example, Phaneuf et al. found that stool samples had the best LOD for all bacteria except *Salmonella*, where urine samples had a better LOD [84]. Leveraging this advantage would allow healthcare providers the freedom to choose the sample sources that are best suited for the target pathogen. Overall, the improvements in this device make it suitable for POC/OS detection while being as reliable as its conventional laboratory counterpart.

While immunoassays for SARS-CoV-2 are less common compared to NA amplification, they are still precise and efficient [85]. Therefore, adapting SARS-CoV-2 as a detection target into a device like the one developed by Phaneuf et al. would not be very difficult, as antibodies for SARS-CoV-2 are available [84]. In addition, the disc contains 20 contained microchannels, each with its own sample inlets and detection sites [84]. Thus, it is possible to test multiple samples from different sources on the same disc without the risk of cross-contamination and improve the testing efficiency.

In an effort to develop an integrated microfluidic device that is capable of high-throughput quantitative amplification, Yeh et al. reported the self-powered integrated microfluidic point-of-care low-cost enabling (SIMPLE) chip [86]. The SIMPLE chip uses isothermal recombinase polymerase amplification (RPA) in lieu of PCR, hence eliminating the need for thermal cyclers in the device. With the shift of decentralized diagnostic tests from qualitative “yes-or-no” answers to definitive quantitative results, digital amplification was chosen for a more informative diagnostic test [86].

**Figure 6:**
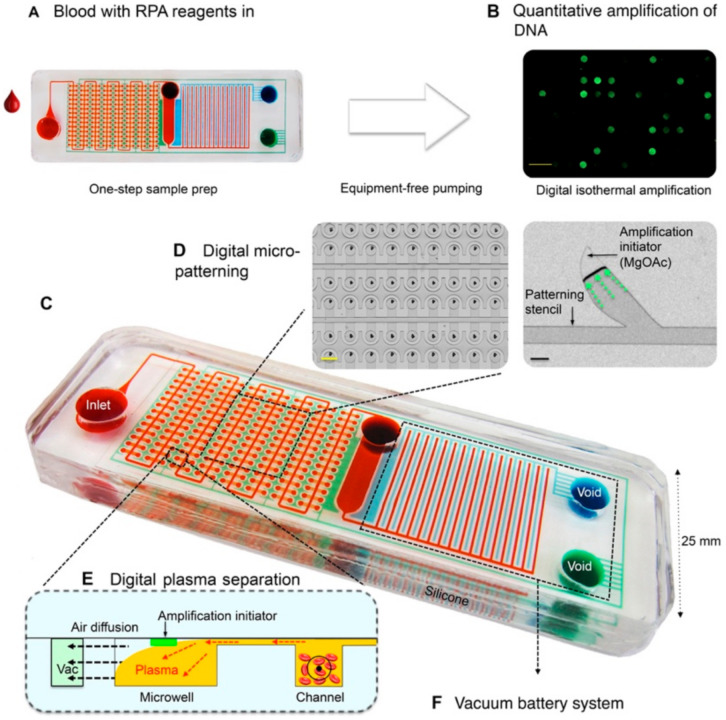
A schematic representation of (**A**) the plasma separation of the sample step, (**B**) the quantitative amplification of DNA through RPA, (**C**) a SIMPLE chip loaded with dye to show the microchannels, (**D**) digital micropatterning after chip bonding (left) and the stencil for the amplification initiator (right), (**E**) the sideview of the on-chip plasma separation setup and (**F**) the vacuum battery system that supplies the SIMPLE chip. Reprinted with permission from ref. [86]. Copyright 2017 AAAS.

The SIMPLE chip shown in Figure 6 is made of two layers of polydimethylsiloxane (PDMS) and was reported to have the capacity to be stored and transported indefinitely inside airtight aluminum pouches [86]. The storage and transportation capabilities of microfluidic-based devices, such as this SIMPLE chip, reinforce the necessity for microfluidic integration in diagnostic tools as a means to augment the accessibility and lifespan of rapid diagnostic tests [86]. Integrated microfluidic devices, such as the one shown in Figure 6, boast improved robustness, as the reagents and necessary components to operate these chips, such as the RPA amplification initiator magnesium acetate (MgOAc), are often prepatterned and loaded into the chip prior to use [86]. For example, the magnesium acetate used for detection in Figure 6′s SIMPLE chip is automatically patterned with a reusable stencil into 2 nL discrete islands onto one of the PDMS layers, while the other layer is pretreated with oxygen plasma to create a hydrophilic surface [86]. The magnesium acetate will adhere indefinitely after the layers are stacked together, allowing for downstream digitization of the results and signal detection from human blood spiked with human immunodeficiency virus 1 (HIV-1) RNA, with a limit of detection as low as 10 copies μL^−1^ in 18 min [86]. Thus the fabrication and replicability of these devices are better facilitated through the integration of microfluidics in order to increase their effectiveness in on-site settings.

In addition to improvements in fabrication, replicability and robustness, on-chip sample preparation can be achieved through the innovative design of the microfluidic channels. One example of this innovative microfluidic design can be seen in the plasma preparation that is performed by SIMPLE chips [86]. In some cases, such as in Figure 6, this is done by including microcliff structures, where an abrupt change in channel height allows for the skimming of blood plasma to effectively separate it from the undesired components in the channels below [86]. Such a simple feature in fully integrated microfluidic devices, such as this, allows for autonomous separation of whole blood in order to prevent hemolysis, which often occurs with mechanical separation [86]. Moreover, alternative high-efficiency amplification methods, such as RPA, can be utilized as the amplification method in SIMPLE chips, as it does not require thermal cycling like PCR does and is more robust in processing plasma samples compared to LAMP [86]. In addition to allowing for alternative amplification methods, fully integrated microfluidic devices offer another advantage in their thermostability. Methods such as RPA can operate in a wide range of temperatures, often 25 to 42 °C, but normally do not have on-site thermostable platforms that can operate effectively at varying temperatures. The temperatures can vary as a result of environmental conditions, or more importantly, based on the type of reaction being performed. Therefore, heightened thermostability allows for greater modularity and flexibility when it comes to the types of reactions and indicators that can be utilized in SIMPLE chips, again highlighting the importance of microfluidic integration.

SIMPLE chips are, therefore, shown to excel regarding the sample preparation and automation pillars of microfluidic integration [86]. It is one of few types of devices that are able to conduct on-chip plasma separation, as most devices rely on off-chip, pretreated blood samples to retrieve plasma samples. The innovative design of the microfluidics in the chip, along with the pretreatment and preloading of necessary reagents for amplification, allow for the assay to be initiated once the sample has been loaded onto the chip. This fully realizes the “sample-in-answer-out” model, as chips like these can operate completely autonomously. SIMPLE chips, including fabrication, materials and reagents, can cost less than 10 USD per device and can achieve full sample analysis within 30 minutes [86]. Moreover, the use of nanoliter microchannels can enhance the assay’s limit of detection, while the inherent nature of digital amplification puts the SIMPLE chip in the high-throughput category as it is capable of parallel processing hundreds of microwells [86].

Similar to the ZIKV paper-based microfluidic device, the SIMPLE chip performs on-chip NAAT [77,86]. While both report quantitative data on the target, the SIMPLE chip is able to provide more concrete information for the quantification of the target due to the digitization of RFA [77,86]. SIMPLE chips were able to detect HIV-1, which belongs to the retrovirus family and has positive-strand RNA, just like SARS-CoV-2 [86,87]. RPA is a technique that is already used for NA amplification in SARS-CoV-2, with primers designed for the RdRp and nucleoprotein genes [88,89]. Therefore, it should not be too difficult to translate SARS-CoV-2 from a laboratory setting onto a microfluidic chip that can be deployed for POC/OS detection. On-chip plasma separation is also an excellent feature that could be adopted into POC/OS testing for SARS-CoV-2. SARS-CoV-2 RNA is found in the blood of some infected individuals, and while blood is not as common as nasopharyngeal swabs regarding testing, it is more commonly found in those who are asymptomatic [90]. This is important, especially when fighting the COVID-19 pandemic, as up to 45% of those who are infected are asymptomatic but are still capable of spreading the virus to others. Higher percentages of asymptomatic infections fuel the pandemic, and therefore it is very important that these asymptomatic cases are detected before they can be spread [91]. With the built-in microcliff structures found on the SIMPLE chip, fast and convenient on-chip plasma separation can be facilitated, allowing for whole blood to be used directly as the testing sample [86]. This further shortens the assay time and allows for rapid detection.

Despite being able to address many of the pillars of integrated microfluidic devices, currently, many of the fully automated devices have yet to achieve 100% automation and high detection accuracy [92,93]. Since devices like the Zika device can rely heavily on capillary action to pull reactants through microchannels, automation is not a large concern [77]. However, full automation in devices that utilize capillary action often lacks modularity that is managed by control chambers, as well as high-control reproducibility, possibly resulting in inconsistent testing, due to the on-site fabrication of these devices. It is possible that by including a large number of assay channels on devices that utilize capillary action, it is feasible to use some channels as quality control channels that provide concrete confirmation on the functionality of the device.

The aforementioned IMPs are examples of diagnostic devices that can offer a variety of specificity in several different mediums of patient samples, such as whole blood, blood plasma and saliva, in ultra-low volumes. These devices, through microfluidic integration, gained the sensitivity to detect pathogens in concentrations as low as 1 unit/mL and nucleic acids as low as 10 to 10^5^ copies/µL [77,84,86,94]. Moreover, one of the most important benefits that resulted from partial or full microfluidic integration was the increase in the turnaround time. Several of these devices were shown to provide accurate results in as little as 10 min, though they can average around 30 min. The improvement in rapid testing, sensitivity and selectivity gained from microfluidic integration is something that these devices can take advantage of in the clinical translation of these diagnostic tools from general pathogens to on-site SARS-CoV-2 testing [95,96].

## 4. Clinical Translation of Integrated Microfluidic Devices for Detection and Quantification of SARS-CoV-2

Current microfluidic devices for the detection and quantification of SARS-CoV-2 in patient samples hold great promise for future on-site diagnostic use. The increase in detection speed, specificity and sensitivity achieved through microfluidic integration largely improve the potential use of these devices in low-resource settings where access to diagnostic tests is severely limited [97,98]. However, many of these techniques are limited by the cost of fabrication in mass production, as well as in their robustness and durability outside of laboratory settings. The hope is that with modifications, these different devices and techniques might be able to eventually achieve microfluidic integration that can be used on-site and reliably diagnose patients infected with SARS-CoV-2.

In Table 2, we present several notable characteristics of four of the most promising SARS-CoV-2 detection technologies currently available. The listed technologies have shown significant advancements in their potential for clinical translation through the integration of microfluidics. One example of note is the work of Qu et al. on microflow cytometry [99]. Despite not currently existing on a mobile platform, the low detection limit, extremely short total assay time and the relatively low cost of this technology demonstrates the effectiveness of novel microfluidic-based integrated diagnostic devices for detecting SARS-CoV-2 in extremely small sample volumes [99]. Although many of the conventional detection methods, such as immunoassay and RT-PCR, have comparable specificity, sensitivity and short turnaround time as a result of microfluidic integration, it does not compare well to other more novel methods. Detection methods such as microflow cytometry and nanoparticle-based detection may offer more of an inherent advantage to SARS-CoV-2 diagnosis solely based on factors such as detection limit, sample volume and quantitative capabilities; however, as previously mentioned, an optimal on-site integrated microfluidic diagnostic device for SARS-CoV-2 detection must adhere to the ASSURED standards set out by the WHO and the seven pillars of microfluidic integration to the best of their ability [39]. Therefore, microfluidic integration appears to hold the most promise in facilitating the clinical translation of our currently existing technology toward a cost-effective, rapid and selective diagnostic tool for SARS-CoV-2 that will be more readily accessible to people from low-resource areas, as well as other parts of the world.

### Stage-Wise Implementation of Microfluidic SARS-CoV-2 Detection

The use of IMPs in SARS-CoV-2 detection can be divided into two categories, with different target populations for each. While a single type of device operating on a “one-size-fits-all” principle could suffice for all SARS-CoV-2 detection, having this differentiation would increase the testing efficiency and the distribution of resources between the two. There are minute differences between healthy, symptomatic, asymptomatic and pre-symptomatic populations that would warrant changes in the device, testing mechanism or sample preparation that are better suited for the intended goal of the diagnostic test [23].

The first type is general testing, which would be used in the screening of healthy people and those who are infected but are pre-symptomatic or asymptomatic. Currently, with over 500,000 SARS-CoV-2 cases confirmed every day and testing positive rates between 0 and 50%, a conservative estimate for the number of tests required each day is well into the millions [3,101]. Having a low cost per test is highly beneficial to ensure the accessibility of these tests, especially for low-income communities. As discussed before, this can be achieved through reducing material costs, such as using a μPAD fabricated with wax printing. Next, a shortened turnaround time for a screening test is also favored. There are two types of SARS-CoV-2 tests available in Canada, with the Abbott Panbio™ COVID-19 Rapid Antigen Self-Test being in closer relation to general screening tests. This qualitative POC device tests for SARS-CoV-2 from a nasopharyngeal swab in a lateral flow assay and can be completed within 15 min [102,103]. Since a qualitative answer could suffice in a general screening test, an IMP developed for general SARS-CoV-2 screening may report its results qualitatively or through simple quantitative colorimetric results using continuous flow microfluidics to conserve time and resources. A turnaround time of 15 minutes is necessary, as longer turnaround times would delay self-isolation and increase the risk of transmitting the virus to others. Therefore, it is in the best interest of public health that any IMPs developed for SARS-CoV-2 general screening tests meet these criteria.

The second type is specific testing and will be geared toward those who are infected and are symptomatic. The pandemic has been ongoing for 20 months, and most people are now familiar with the flu-like symptoms of COVID-19. Those who experience these symptoms are more likely to seek a SARS-CoV-2 test to determine whether they have been infected. In Canada, a qRT-PCR test is performed to confirm the presence of the virus and determine the viral load. A nasopharyngeal sample is taken and sent to a government laboratory, with results available in one to three days. This is significantly longer than the Abbott Panbio™ Rapid Test and can be improved with the use of IMPs. Many microfluidic platforms that perform quantitative NAAT on viral pathogens report a turnaround time of within an hour, as seen in the ZIKV device and the SIMPLE chip [77,86]. Shortening this turnaround time will curb the spread of the disease. Moreover, a quantitative test is preferred in this type as the viral load, which correlates to the transmissibility and severity of the virus, can assist healthcare providers in determining suitable treatment plans for patients [104,105]. Thus, IMPs developed for specific SARS-CoV-2 testing should meet these criteria, potentially through the use of droplet microfluidics for quantification.

Serological tests, which test for anti-SARS-CoV-2 IgG and IgM antibodies, are available to test whether a person was previously infected or had gained antibodies through COVID-19 vaccinations [106]. However, with SARS-CoV-2 and its variants fueling serious waves of new infections globally, more focus should be put into tests that determine whether someone has the virus at the time of testing, such as the ones mentioned before.

## 5. Conclusions

In this review, we discussed the fundamentals of conventional diagnostic testing platforms, currently existing platforms and the potential applications of integrating microfluidics toward achieving reliable and accessible on-site diagnostic testing devices. The inability of conventional diagnostic testing tools to meet the unmet demand for safely, cheaply, and reliably mass-producing accurate diagnostic tests for these devices can be seen as a limitation to controlling and monitoring the spread of highly infectious viral infections, such as SARS-CoV-2. New technologies, such as complete or partial microfluidic integration, can be used to overcome several of the limitations that are related to conventional diagnostic tools, such as the need for access to expensive laboratory equipment for sample processing. Integrated microfluidic-based diagnostic platforms have the ability to screen for and analyze patient samples in a more realistic setting without sacrificing accuracy or robustness. For example, the use of partial microfluidic integration has already shown effectiveness in diagnosing ailments, such as cancer and infectious diseases, that are not transmissible through respiratory pathways, such as the Zika virus [104]. Additionally, near or fully integrated microfluidic platforms showed that they can effectively test for viral DNA/RNA under a wide range of conditions using a self-contained and versatile SIMPLE chip for both qualitative and quantitative analysis. Furthermore, in each case of microfluidic integration, the capacity for on-site diagnostic potential has increased. With microfluidic integration, any of the seven pillars of microfluidic integration effectively shortens the gap between unviable on-site diagnostic tests and fully realized on-site diagnostic tests. Recent advances in this continuously expanding field show strong promise in the reduction of the cost per device, the increased accuracy and modularity, the robustness, and most importantly, the speed of the sample analysis.

Nevertheless, there remain many key obstacles in facilitating the transition of our currently available diagnostic tests to mass production, as discussed in the criteria set out by this review. The WHO’s ASSURED criteria, which defines the minimum standards for developing on-site medical diagnostic tests, can be easily achieved through microfluidic integration. For example, the global cost of producing such on-site diagnostic devices can be greatly reduced through downsizing to the microscale and the development of self-contained devices. This would be an important feature to address with microfluidic integration before the benefits outweigh the costs to mass-produce these on-site diagnostic tests to meet the current global demand. Though global costs can be reduced as more microfluidic mediums are being discovered, device sensitivity can still be limited by the diagnostic technology that is currently available to detect certain pathogens. For example, despite the spike protein of SARS-CoV-2 being well documented, there remains room for improvement in the specificity and efficiency of indicators and markers to facilitate further diagnostic improvements. Microfluidic integration can maintain the level of accuracy shown in their non-microfluidic diagnostic counterparts but will require greater feats of innovation before significant improvements to the accuracy of on-site detection methods can be achieved. Maintaining, or improving, the accuracy of IMPs is just one example of the “seven pillars” for assessing effective IMPs we have developed. On-site diagnostics of infectious pathogens, such as SARS-CoV-2, should look to a combination of the ASSURED standards and our “seven pillars” criteria to productively work toward an effective on-site diagnostic tool. This review details the basic and advanced information regarding microfluidics and the benefits of microfluidic integration in the detection and quantification of several infectious pathogens. Using SARS-CoV-2 as a current and relevant example, we developed a framework to take our findings on microfluidic integration and translate them toward the development of an effective on-site diagnostic tool for detection and quantification.

Efficient multiplexing and parallel testing so that each test can detect more than one specific pathogen is a characteristic of current on-site IMPs that has yet to be achieved in a cost-effective manner. A significant trend in developing integrated microfluidic diagnostic devices is the integration of multimode biosensor technologies to optimize data analysis and collection. Integrated microfluidic devices, combined with multimode sensing and improved algorithmic analysis, can create systems where real-time digital processing can be achieved while analyzing samples for multiple targets. In addition, there were recent improvements in droplet microfluidic technology that make use of these multimode biosensors to allow for multiplexing and single-cell analyses. Therefore, we envision that integrated microfluidic devices can achieve improved diagnostic capabilities with greater accuracy and improved detection limits with multimode sensing and improved real-time analysis software.

## Figures and Tables

**Table 1:** Comparison of the microfluidic devices based on the most commonly used type of material [36,57].

	Glass	Silicon	Polymer	Paper
**Fabrication Techniques**	Photolithography Etching	Bulk or surface micromachining Nano-imprint lithography Electron beam irradiation	Soft lithography Injection molding 3D printing	Wax and inkjet printing Photolithography
**Advantages**	Transparent Inert and stable Solvent compatible Hydrophilic	Mechanically strong Thermostable Chemical resistance	Transparent Easy fabrication Low cost	Flexible and lightweight Low cost No need for external pumps or valves Biocompatible Recyclable
**Limitations**	Brittle Not flexible High cost	High cost Biocompatibility	Hydrophobic Short shelf life	Humidity and temperature sensitive Difficult to design and integrate into single chip

**Table 2:** Comparison of several recent advancements in SARS-CoV-2 detection methods and their future on-site diagnostic potential for SARS-CoV-2.

	Immunoassay	RT-PCR	Nanoparticle	Microflow Cytometry
*Reagent* *Consumption*	10 µg (in tube)	20 µL (in tube)	Negligible	50 µL (in tube)
*Target of Detection*	IgG, IgA, IgM	N gene, E gene	Gold-spiked	IgM, IgG
*Limit of Detection*	0.15 mg/L	1-10 copy per µL	0.08 mg/L	0.06-0.10 mg/L
*Total Assay Time*	1 h	2 h	2–5 h	30 min
*Sample Volume*	20 µL	120 µL	1 µL	10 µL
*Assay Control*	Automated	Manual	Manual	Automated
*Cost per Test*	~ 6 (USD)	~ 4 (USD)	~ 10 (USD)	~ 5 (USD)
*Quantitative*	No	Yes	Yes	Yes
*Mobile*	Yes	Yes	No	No

Disclaimer: the cost analysis approximates the cost for the materials and reagents required to make one testing device based on a mix of reported material costs and independent research into the materials used [12,98,99,100].

## Data Availability

The data presented in this study are available upon request.
